# Ileocolic intussusception due to Burkitt lymphoma: a case report

**Published:** 2013-03-25

**Authors:** NR Bălănescu, L Topor, D Malureanu, I Stoica

**Affiliations:** Department of Paediatric Surgery, “Grigore Alexandrescu" Clinical Emergency Hospital for Children, Bucharest, Romania “Carol Davila" University of Medicine and Pharmacy, Bucharest, Romania

**Keywords:** ileocolic intussusception, Burkitt lymphoma, ileal tumor, termino-terminal anastomosis

## Abstract

Burkitt lymphoma has many forms of clinical presentations and, in children, it is usually discovered due to the presence of an abdominal mass. This rapidly growing tumor is highly malignant, aggressive, and may cause either indirect symptoms, due to pressure phenomena or direct involvement of the bowel lumen, leading to either intestinal obstruction or intussusception.

We describe the case of a 4-year-old girl who exhibited an unusual presentation of ileocolic intussusception on a Burkitt lymphoma lesion of the ileum.

## Introduction

Burkitt lymphoma represents 8-10% of all tumors in children under 15 years of age [**1**]. First described by Dennis Burkitt in 1958 [**2**], Burkitt lymphoma is an aggressive, highly malignant and rapidly growing B-cell neoplasm, frequently presenting with onset in the abdomen and found in non-endemic Burkitt lymphoma regions [**3**]. 

 This type of lymphoma has three clinical forms. The endemic form, common in the African continent, develops due to a chromosomal translocation between chromosomes 8 and 14 and often presents as a rapidly growing tumor of the facial bones. The non-endemic form, observed most often in the United States, typically presents with an abdominal mass or ascites. This non-endemic form results from a chromosome 8 translocation involving the c-myc oncogene. The third form is specific to immunocompromised patients and frequently presents as diffuse lymphadenopathy [**4,5**]. 

 Burkitt lymphoma has a high proliferation rate and the histological appearance of a “starry sky" because of the high rate of mitosis and spontaneous cell death [**2,6**].

 Intussusception caused by Burkitt lymphoma as a cause of acute abdomen is rare, with often misleading symptoms that make the diagnosis more difficult [**3**]. Many pediatric patients with Burkitt lymphoma present with intussusception as a first clinical sign, a presentation that potentially leads to the disease detection at an earlier stage [**7**].

 Children with Burkitt lymphoma have an excellent prognosis with contemporary treatment regardless of disease stage [**7,8**].

## Case report

We present a case of a previously healthy 4-year-old girl who was admitted in our hospital with blood in the stool for the previous month, associated with loss of appetite and significant weight loss of 3 kg. In the 24 hours prior to admission to our surgical emergency clinic, the child complained of vague abdominal pain and had fever (38.2 ˚C). On admission, the child was pale and sweaty, with a distended, diffusely tender and painful abdomen and with normal-consistency stools mixed with blood. The patient was anemic, with a hemoglobin level of 8.6 g/dl, a hematocrit of 22.9 % and an ESR of 45 mm/h. She was hypoproteinemic (TP=5.9 g/dl) due to hypoalbuminemia. The stool sample showed no pathogenic bacterial growth.

 Ultrasonography examination of the abdomen showed a 5.3/3 cm tubular image on longitudinal section with a “coiled spring" appearance in the right upper quadrant.

 On CT scan, (abdomen and pelvis, with and without contrast), we observed a solid, heterogenous tumor mass of 5.4/3 cm, with an irregular contour located in the right flank. The appearence was similar to that of a “pseudo-kidney" or “target sign" and appeared to be attached to the right colon; the small intestine loops were dilated, with a thick wall and a few air-fluid interfaces located in the inferior abdomen.

**Fig. 1:**
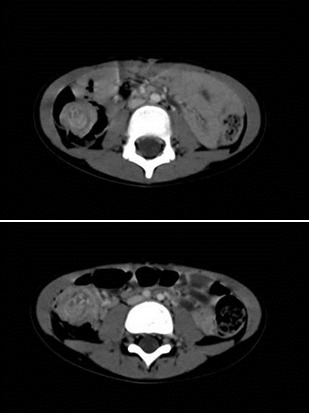
Aspects of CT scan at admission

 Colonoscopic examination revealed no tumors in the lumen of the examined segments, but there were some stool fragments containing both digested blood and mucus ("red currant jelly" appearence).

 After the stabilization and correction of electrolyte imbalances, a laparotomy was performed under general anesthesia with oro-tracheal intubation. The patient was placed in the supine position, and a midline laparotomy was performed, which revealed an ileo-colic intussusception (terminal ileum, cecum and ascending colon) that was easily reduced; this procedure revealed the presence of a tumor in the terminal ileum, located 10 cm from the ileocecal valve, measuring approximately 5 cm in diameter and with a firm consistency, that occupied almost all of the lumen and invaded the antimesenteric ileal wall.

 We performed a segmental resection of the terminal ileum with excision of the tumor, retaining margins of 5 cm on each side of the tumor, and an ileo-ileal end-to-end double layer anastomosis; we took ganglionar biopsies from the jejunal, ileal and mesocolic ganglia.

 Postoperatively, the patient had an uneventful recovery and was discharged home on day 7 after surgery.

 The histopathologic examination of the excised tumoral mass proved to be a Burkitt lymphoma; the lymphatic ganglia showed only signs of chronic reactive lymphadenitis. 

**Fig. 2:**
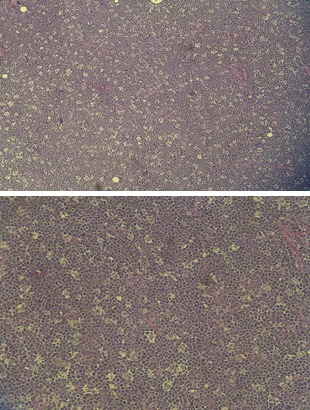
Histopathological appearance

 According to St. Jude’s staging system, we classified our patient as a stage II Burkitt lymphoma, and we referred her to the oncology department for evaluation and initiation of the chemotherapeutic treatment. She was treated with cyclophosphamide, adriamicyn, vincristine and prednisolon for 2 courses. 

 Eighteen months after surgery, our patient still had not relapsed – clinically or ultrasonographically; she exhibited good treatment compliance and followed her oncologist’s recommendations to the letter.

## Discussion

Intussusception is one of the most common emergencies in children that require the attention of a pediatric surgeon. Especially in older children, in 10% of the cases, there is a pathological lesion (Meckel’s diverticulum, polyp, and lymphoma) at the lead point of the intussusceptum [**7,9-11**].

 Although 90% of intussusceptions occur in children between 3 months and 3 years of age, with a peak incidence between the fifth and the ninth month of life, intussusception has also been reported in utero, in neonates and in adults [**9,11**]. Estimated incidence is 2-3 cases per 1000 live births with boys/girls sex ratio 3:2 [**7,11**].

The classic clinical triad described for intussusception is made up of abdominal colic, “red currant jelly" stools and a palpable abdominal mass; in children older than 2-3 years the presentation of intussusception is subtler and the classic triad of symptoms may not be present [**9**]. 

 Ultrasonography is the most efficient examination for the diagnosis of intestinal intussusceptions with a reported accuracy of up to 100% [**9**], and combined with clinical observation, is usually sufficient to diagnose the intussusceptions [**11**]. Barium enema, CT and colonoscopy can also be helpful in diagnosis [**12**].

Primary gastrointestinal lymphoma represents 1%-4% of all gastrointestinal malignancies. Most cases in children involve the distal ileum or ileocecal region [**13,14**]. Non-Hodgkin’s Lymphoma (NHL) represents the third most frequent cancer in childhood and usually is subdivided into 3 histological subtypes: (1) 65% B-cell NHL including both Burkitt lymphoma and diffuse large B-cell lymphoma; (2) 20% lymphoblastic and (3) 15% anaplastic large cell lymphoma [**15,16**]. Burkitt lymphoma represents 40%-50% of all NHL cases in childhood, and is more common in males than females [**3,11-14**]. Predisposing factors for lymphoma of the small intestine includes prior malabsorbtion syndromes, inflammatory bowel disease and immunodeficiency states [**14**]. All these risk factors were absent in our patient who were previously healthy.

 The clinical presentation of undiagnosed Burkitt lymphoma is non-specific, which makes this a difficult condition to diagnose. Abdominal pain is present in 80% of the cases of intussusception with Burkitt lymphoma, along with nausea, vomiting, constipation, diarrhea, fatigue or malaise [**7,18**]. The rapidity of volumetric doubling of this neoplasm frequently leads to an acute abdomen presentation that may mimic other diseases (bowel obstruction secondary to ileocecal intussusception caused by tumor growth, obstruction or bleeding; some presentations may mimic acute appendicitis) [**3,12**]. In the case we are presenting, the onset was slow, with the patient losing weight and, prior to admission, showing the following two of the three classic signs for intussusception: diffuse abdominal pain and bloody stools without any palpable abdominal mass.

 The ideal treatment of gastrointestinal lymphoma must be individualized based on the type of disease and its location so that a multidisciplinary approach with surgery and chemotherapy can optimize the chance of cure [**13-15**]. Along with the assignment of specific treatment protocols of relatively short duration, event-free survival rates for children with B-cell NHL have dramatically increased within the last 20 years [**15**]. Surgical procedures are now needed for complete resection in limited disease, diagnostic biopsies, management of life-threatening local tumor effects and second-look operations [**15**]. Surgery may provide important prognostic information through definitive staging offers a chance for cure with or without adjuvant therapy and may prevent complications such as haemorrhage, obstruction or perforation [**13**]. In patients with proven localized abdominal disease total resection may be performed if is not mutilating (as hemicolectomy performed in the past). The open surgical approach is still the gold standard in most paediatric surgical facilities [**11**]. Patients with complete resection had a significantly better prognosis compared with patients with incomplete resection or biopsy [**15**].

 Burkitt lymphoma is very sensible to chemotherapy [**19**], therapy courses include the following drugs: cyclophosphamide, methotrexate, cytarabine, iphosphamide, etoposide, vincristine, vindesine, adriamycin, doxorubicin, dexamethasone [**8,15**]. Burkitt lymphoma patients who present with intussusception require shorter-duration and less intense chemotherapy than patients diagnosed in other ways. In our patient, we performed a segmental resection of the terminal ileum with total resection of the tumour, 2 courses of chemotherapy with excellent outcome at 18 month after surgery. 

## Conclusions

Burkitt lymphoma is a diagnostic entity that the pediatric surgeon should consider when faced with a child with an unspecific clinical presentation, who complains of diffuse abdominal pain, weight loss, and bloody stool (with a “red currant jelly" appearance). The suspicion of intestinal intussusception with a pathological lesion at the lead point of the intussusceptum necessitates surgery. Laparotomy is the gold standard in both diagnosis and treatment, ensuring the excision of the entire tumor with free margins. A multidisciplinary team with an oncologist assures efficient therapeutic management. Short- and long- term outcomes are generally favorable, as shown by data from a review of the literature and confirmed by our patients’ outcome.
